# MR1, an immunological periscope of cellular metabolism

**DOI:** 10.1093/intimm/dxab101

**Published:** 2021-10-27

**Authors:** Andrew Chancellor, Alessandro Vacchini, Gennaro De Libero

**Affiliations:** Experimental Immunology, Department of Research, University of Basel and University Hospital, Basel, Switzerland

**Keywords:** antigen presentation, MAIT, metabolite antigens, MR1T cells, tumour metabolites

## Abstract

The discovery that major histocompatibility complex (MHC) class I-related molecule 1 (MR1) presents microbial antigens to mucosal-associated invariant T (MAIT) cells was a significant scientific milestone in the last decade. Surveillance for foreign metabolically derived antigens added a new class of target structures for immune recognition. The recent identification of a second family of MR1-restricted T cells, called MR1T cells, which show self-reactivity suggests the microbial antigens characterized so far may only represent a handful of the potential structures presented by MR1. Furthermore, the reactivity of MR1T cells towards tumours and not healthy cells indicates tight regulation in the generation of self-antigens and in MR1 expression and antigen loading. These novel and exciting observations invite consideration of new perspectives of MR1-restricted antigen presentation and its wider role within immunity and disease.

## Teaser

MR1-retricted T cells in health and disease

## Introduction

The immunology of major histocompatibility complex (MHC) class I-related molecule 1 (MR1)-restricted T cells is a rapidly expanding field, principally because of an increasing number of discoveries related to both the biology of MR1 as an antigen-presenting molecule and the biology of MR1-restricted T cells. From the seminal discoveries of a gene encoding an MHC class I like protein (1–3), much effort has been invested in the discovery and characterization of MR1-presented antigens and MR1-restricted T cells. This fascination is due in part to the fact that MR1, like CD1 molecules, presents non-peptidic antigens, illustrating the enormous flexibility of T cells to recognize many different chemical structures. Furthermore, the MR1 gene is oligomorphic, underlying their potential use in MR1-restricted T cells in cell therapy. MR1 is also ubiquitously expressed by human tissues (3) and the human protein shares a 90% identity with its murine ortholog (2), indicating a strong evolutionary pressure on its structural properties (4).

## MR1-restricted T cell families

The biological relevance of MR1 has been clarified with the attribution of antigen presentation to the previously characterized innate-like population of mucosal-associated invariant T (MAIT) cells (5–8). More recently, a second family of MR1-restricted T cells, called MR1T cells, has been identified. MR1T cell clones identified so far do not respond to the same microbial antigens as MAIT cells, are self-reactive and preferentially recognize tumour cells (9, 10). Such findings are expanding the field of MR1 to include the exploitation of this molecule for tumour cell targeting in cancer immunotherapies. The main immunological characteristics of MAIT cells have been exhaustively reviewed elsewhere (11–14); we therefore discuss current knowledge of the MR1-restricted T cell repertoire.

MAIT cells have emerged as important players within the immune response because of their anti-microbial activity at barrier sites. MAIT cells are one of the most abundant antigen-specific populations of T cells in healthy individuals and whereas they represent 1–10% of all circulating T cells in blood (15, 16), their numbers are enriched within mucosal tissues, gut lamina propria and the liver (17, 18). Within these tissues, MAIT cells contribute to the maintenance of the integrity of the epithelial barrier and killing of infected cells (17, 19, 20) after recognition of microbe-derived metabolite antigens (8, 21).

The ability of MAIT cells to sense bacterial antigens presented by MR1 is conferred by their semi-invariant T cell receptor (TCR). MAIT cells mostly express an invariant Vα7.2–Jα33 rearranged chain (encoded by the *TRAV1-2* and *TRAJ33* genes). In some instances, other TRAJ genes are used (*TRAJ16* and *TRAJ20*), which maintain a CDR3α loop similar in length and with a conserved tyrosine in position 95, important for antigen recognition (5, 6, 17, 22–24). The TCR β-chains of MAIT cells are oligoclonal and consist mainly of Vβ2 (*TRBV20*) or Vβ13 (*TRBV6*) chains (16, 17, 25–27). Recently, other populations of TRAV1-2^−^/5-OP-RU MR1-tetramer^+^ ‘MAIT-like’ and ‘non-MAIT-like’ populations have also been identified based on the expression of all or none of three MAIT markers CD218a (IL-18Rα), CD26 and CD161, respectively. While ‘non-MAIT-like’ cells and only some ‘MAIT-like’ cells expressed diverse TCRs, another subset of ‘MAIT-like’ cells exclusively expressed a TRAV36^+^ TCR, representing a public TCR (23), although these studies did not verify cell activation with the MAIT antigen 5-(2-oxopropylideneamino)-6-d-ribitylaminouracil (5-OP-RU). A few studies also described rare MAIT-like cells that do not share the conventional phenotype and do not express the Vα7.2 chain, but still recognize MAIT-stimulatory microbial antigens and are therefore named atypical MAIT cells (24, 26, 28).

One classical MAIT cell TCR previously described was also unusual in its ability to maintain reactivity to 5-OP-RU despite the introduction of a Y95F mutation in the α-chain (24). During infection, TRBJ usage and N-nucleotide additions within the CDR3β loop were associated with differential clonotypic recognition of antigens and bacterial species (25, 29). The minor alterations in the length, flexibility and MR1–antigen contacts of the CDR3β loop consequentially drive the MAIT cell repertoire after infection, where clonal expansion occurs in those with higher functional avidity (25, 30). More importantly, here, is that the β-chain repertoire of MAIT cells determines their functionality and further investigation may reveal additional antigen specificities.

In contrast, MR1T cells, so far only studied in humans, constitute a separate family of MR1-restricted cells, since they do not react to the prototypic microbial antigens and appear to have a phenotype distinct from that of MAIT cells. MR1T cell clones have been identified by their ability to react to self-antigens presented by tumour cells and other healthy cells in an MR1-restricted manner (9, 10). The frequency of MR1T cells in the blood of healthy donors is much lower than that of MAIT cells, similar to the frequency of conventional antigen-specific T cells (9). They are heterogeneous, have diverse TCR gene usage and frequently express the CD8 co-receptor (9). The functional analysis of several different MR1T clones revealed a broad spectrum of functions, typical of Th1, Th2 or Th17 cells. Indeed, isolated MR1T cells secreted different cytokines or other soluble molecules and showed distinct transcriptional profiles upon antigen recognition (9).

The heterogeneity of these cells is reflected by the diverse immune activities in which they participate, that in addition to tumour cell recognition support of anti-microbial activity by endothelial cells and promotion of dendritic cell (DC) maturation (9). One MR1T cell was found to react to several tumour cell lines and not to human healthy cells (10). Although the involvement of TCR-mediated recognition of each tested tumour cell line was not investigated, these data confirmed previous data (9) suggesting that the occurrence of MR1-presented antigens can be shared by several tumour cells.

## The repertoire of antigens presented by MR1

The variety of responses and functional activities exerted by MR1-restricted cells can be explained by the breadth of antigens that MR1 can present. MR1 binds antigens inside a pocket located atop a β-pleated sheet, surrounded by two α-helices contributed by the α1 and α2 domains, respectively (8). The antigen-binding pocket is divided into two communicating pockets, called A′ and F′, respectively. They form a large cavity overlooked by aromatic amino acids that define a hydrophobic and charged space where antigens based on small cyclic and bicyclic molecules are fitted and presented for direct interaction with MAIT TCRs (8, 31). As it is closed at its ends, in contrast to MHC class II, the MR1 pocket might appear to have limited tridimensional space for antigen hosting (8, 21, 31) but, given the nature of the ligands characterized so far and considering the vast chemical diversity occurring among small molecules, it is very probable that the MR1 pocket can be occupied by an extremely broad spectrum of different molecules. This is also inferred by the variety of masses of compounds eluted from MR1 (28).

Vitamin B metabolites were the first MR1 ligands that were discovered. The precursor of bacterial riboflavin (vitamin B2), 6,7-dimethyl-8-d-ribityllumazine (RL-6,7-diMe), is an antigenic ligand of MR1, able to induce the activation of MAIT cells (8). A metabolite of dietary folic acid (vitamin B9), 6-formylpterin (6-FP), was the second discovered MR1 ligand. This small molecule potently antagonizes the presentation of bacteria-derived antigens to MAIT cells.

Another study showed that the riboflavin precursor, 5-amino-ribityl amino uracil (5-A-RU), generates the unstable adducts 5-(2-oxoethylideneamino)-6-d-ribitylaminouracil (5-OE-RU) and 5-OP-RU upon combination with glyoxal and methylglyoxal, respectively (21). These two adducts form a Schiff’s base with lysine 43 of MR1, stabilizing the metabolites and preventing their conversion into lumazines. The presence of the Schiff’s base is necessary to make these compounds immunogenic to MAIT cells, because it is required to let them remain stably bound to MR1 (21). A consequence of this stability is the much higher potency of both adducts when compared with the previously characterized RL-6,7-diMe (21). The two types of antigens also differ in their capacity to expand populations of MAIT cells expressing different surface markers (32). These discoveries allowed definition of the way in which antigen recognition by MAIT cells occurs and provided unique tools to study MR1 biology, for example by generating MR1 tetramers loaded with 5-OP-RU (21) or generating synthetic analogs, which are useful tools in investigating TCR recognition of MR1–antigen complexes (33).

To further explore the MR1 ligand repertoire, different approaches have been pursued. Mass spectrometry of compounds eluted from soluble MR1 that were produced in the presence of different live bacteria led to the identification of other molecules, still related to the riboflavin biosynthetic pathway, such as 7,8-didemethyl-8-hydroxy-5-deazariboflavin (FO), photolumazine I (PLI) and photolumazine III (PLIII) (28). These studies highlight that other bacterial antigen structures bind MR1 and may stimulate MAIT cells, although their relevance during infection remains to be investigated in *in vivo* models. Another important conclusion is that MAIT TCRs may cross-react with several molecules. The degree and relevance of such cross-reactivity remains to be investigated.

Two other studies relied on computational power to *in silico* dock molecules inside the MR1 pocket. This approach brought about the identification of synthetic compounds that bind MR1, like small molecules and drug metabolites (31, 34). Some of these compounds weakly stimulate MAIT cells, thus confirming the existence of cross-reactive MAIT TCRs.

Furthermore, Salio *et al*. characterized 3-[(2,6-dioxo-1,2,3,6-tetrahydropyrimidin-4-yl)formamido]propanoic acid (DB28), a compound able not only to block MR1 antigen-presenting function, but also to reduce MR1 surface expression (34). This compound, when fed to cells, retains MR1 inside the endoplasmic reticulum (ER) in an immature conformation, in contrast with other inhibitory ligands such as 6-FP, acetyl-6-FP (Ac-6-FP) and the Formylsalicylic acid metabolites 3-FSA and 5-FSA that instead promote MR1 refolding and mobilization from the ER to the plasma membrane (8, 31). Although physiological and functional homologs of DB28 remain to be discovered, these findings show that MR1 protein refolding is sensitive to the presence of different small molecules and some have the capacity to prevent physiological surface appearance. This is unusual for compounds that bind to other antigen-presenting molecules, such as MHC-binding peptides and CD1-binding lipids and could represent a mechanism for pathogen evasion of MR1-restricted T cell recognition.

## Implications of a broad MR1-binding self-antigen repertoire

An important and additional indication that the MR1 antigen-binding capacity is broader than currently appreciated comes from the identification of MR1T cells that recognize tumour cells. The self-antigens that are presented by MR1 that is expressed by tumours or metabolically altered cells have not yet been characterized, but it is already clear that there are likely different groups of antigens, since individual MR1T clones distinguish between different purified antigen fractions and can also differentially recognize distinct tumour cells (9). This allows speculation that MR1 expressed by each different tumour cell type might present a peculiar variety of antigens. These studies also showed that individual MR1T cells may recognize several tumour cell lines, in a non-overlapping manner, thus suggesting a kind of pattern recognition of tumour cells.

A more recent study showed that one MR1T cell clone may recognize a variety of tumour cells (10). Although the MR1-dependent tumour recognition was not confirmed in all instances, this study was in line with those in which recognition of a smaller number of tumour cell lines was investigated (9) and confirmed that MR1T cells can also recognize antigens shared among different tumour cell lines.

All the evidence accumulated so far points towards a model where MR1 can bind a large variety of molecules with different affinities. The microbial compounds identified so far and the indirect evidence obtained with MR1T recognition of tumour cells suggests that MR1 exerts the role of a promiscuous metabolic sensor capable of alerting the immune response. In other words, MR1 can be considered as a molecule capable of surveying the metabolic integrity of cells. This might resemble the role of human leukocyte antigen (HLA) class I molecules that present new peptides allowing T cells to survey changes in the sequence of proteins even if they reside intracellularly. Under physiological conditions, a large fraction of MR1 molecules are retained in the ER, whereas those exported to the surface recycle back and forth over the plasma membrane and present self-antigens or microbial ligands acquired in the ER or in the endolysosomal compartment (35, 36). In the context of an infection, MR1 can be loaded with riboflavin metabolites or other bacterial antigens that are able to induce MAIT cell activation.

**Fig. 1.**
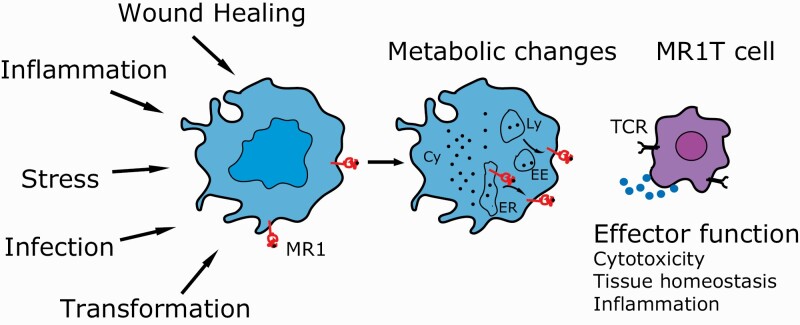
Cellular alterations that may lead to MR1–ligand generation. Professional antigen-presenting cells or other healthy cells such as epithelial cells may undergo stress through different mechanisms. The cells may also be located near to inflammation or physical damage, undergo transformation or proliferate, which leads to an altered metabolism of the cell. The change in composition of metabolites that bind MR1 causes up-regulation to the cell surface, which is registered by MR1T cells to cause an effector function. Cy, cytoplasm; EE, early endosome; Ly, lysosome.

This role, however, is not limited to the accommodation of foreign metabolites. In fact, it is now becoming more evident that MR1 could offer a sample of a cell’s metabolism to MR1T cells, thus recognizing alterations occurring during tumour transformation or other conditions of persistent cell stress (Fig. 1). This may have profound implications in underlying inflammation in many circumstances.

It is unknown how self-antigens contribute to MR1 surface levels. The paucity of surface-expressed MR1 could indicate that, under homeostatic conditions, self-antigens poorly bind MR1, thus preventing their presentation to T cells. A second possibility could be that the low MR1 surface levels are a consequence of limiting amounts of self-antigen produced, as suggested previously (37). Finally, the possibility exists that it is a consequence of limited access of self-antigen to MR1. This latter mechanism would resemble presentation by CD1b of self-phosphatidylglycerol, which in normal cells is present in mitochondrial membranes and cannot access CD1b (38). It is also known that cells differ in the regulation of MR1 expression in response to Toll-like receptor (TLR) signals, possibly via nuclear factor-kappa B (NF-κB) (39). In summary, several mechanisms may regulate the expression of endogenous levels of MR1 and, by implication, the presentation of endogenous antigens. Such regulation may be dependent on antigen type, antigen availability, the presence of microbes, type 1 interferons (IFNs) and the cell type.

Another ongoing question concerns the mechanisms of antigen loading to MR1, which is likely aided by chaperones and possibly a low pH within late endosomes and lysosomes (39, 40). However, since metabolites are not generated in the vicinity of empty MR1 and are spatially separated by different membranes, the question arises of how potential metabolite antigens get from different cellular compartments to the ER, where nascent MR1 folds, or late endosomes, where MR1 molecules recycle from the plasma membrane. The answer has yet to be determined because the antigens are unknown and therefore so are the mechanisms of antigen entry into the ER or endosomes, known only to be transporter-associated with antigen processing (TAP) and protease-independent (40). The active and dynamic maintenance of metabolite levels around the cell could be significant, given that if metabolites are moved towards MR1, the result would be to shift the activation state of responding T cells. Indeed, differential efficiencies in MR1-loading pathways, dependent on an intracellular or extracellular source of antigens, could indicate that some cell types or pathways are preferentially used for endogenous antigen loading of MR1 (35).

To summarize, MR1 is able to bind a number of different molecules that are retained in its pocket by electrostatic interactions or by covalent bonds. These molecules are diverse and appear to have redundant or opposite functions, like RL-6,7-diMe and Ac-6-FP compared with 5-OP-RU, respectively. The flexibility of the MR1 binding pocket is suited to binding synthetic small molecules such as drug metabolites as well as cell endogenous metabolites, many of which are yet to be identified. In addition, indirect evidence of differential MR1T reactivity patterns towards different cell types suggests the presence of many undiscovered MR1-bound antigens.

For the interesting perspective of MR1T cell exploitation in therapy and to better understand MR1 biology, the characterization of MR1 self-antigens remains a most important topic to still be addressed. The first step should reveal the nature of these molecules and disclose the features that make them antigenic to MR1T cells. Are these compounds limited in size as the ligands already described, or are some of them large enough to occupy both the A′ and F′ pockets of MR1? Understanding how these antigens are generated inside cells, i.e. whether they derive from distinct metabolic pathways or if different molecules can arise from common pathways, will also be important. Other important questions are whether they accumulate in all cells, both normal and transformed but in different quantities, or instead are preferentially synthesized in tumour cells.

## MR1-restricted T cells in health and disease

MAIT cells have been shown to recognize microbial antigens; they have also been implicated in several diseases. The contribution of MAIT cells to the resolution of bacterial infection is supported by their location at mucosal sites in both humans and mice, their significant alterations during bacterial infections and their strong response to microbial antigens.

Robust MAIT cell activation occurs through the TCR that is able to detect bacterially infected cells via interaction with MR1 complexed to bacterial antigen. Such recognition of infected cells has been described on many occasions and towards different bacteria such as *Escherichia coli* (19), *Mycobacterium tuberculosis* (35, 41), *M. bovis* (17, 20), *Legionella longbeachae* (42), *Helicobacter pylori* (43, 44), *Streptococcus pyogenes* (26) and *Francisella tularensis* (45). The interaction of MAIT cells with *M. bovis*-infected target cells also leads to killing of intracellular mycobacteria (17), thus emphasizing the protective capacity of MAIT cells during infection.

Innate receptors also heavily influence MAIT cells. For example, MAIT cell accumulation in the lung was dependent on riboflavin-derived antigens in addition to innate TLR2, TLR3, TLR6 and TLR9 ligand engagement (46). Furthermore, TCR-independent activation of MAIT cells occurs through cytokine receptors, primarily interleukin-12 (IL-12) and IL-18 (47) but also interferon-gamma (IFN-γ), tumour necrosis factor-alpha (TNF-α) and IL-1β, and also has profound influence on MAIT activation, altering their response in the presence or even absence of antigen (13). In the liver, TCR-mediated activation of MAIT cells is licensed by IL-7 released by hepatocytes during inflammation (48), indicating the important contribution of the local microenvironment. The combination of activation signals endows MAIT cells with rapid, sensitive and wide-ranging detection of bacteria and inflammation.

MAIT cell functions towards bacterial antigen are primarily pro-inflammatory and cytotoxic, but their functions are diverse. In another model, the absence of MR1 and therefore MAIT cell accumulation in the lungs led to fatal infection (49). Mechanistic studies in a mouse model of *F. tularensis* mucosal infection demonstrated that MAIT cells also have a role in maturation of DCs via recognition of microbial antigen presented by MR1 and release of granulocyte macrophage-colony stimulating factor (GM-CSF) (45). In addition to bacterial detection, appreciation is growing for the role of MAIT cells in other diseases and in homeostasis such as their role in maintaining barrier tissues. Transcriptional signatures for tissue regulation have been identified (50, 51), suggesting that bacterial recognition confers barrier protection. Further, in mouse models MAIT cells preferentially located in the dermis of skin and promoted wound healing, an effect that was enhanced upon topical application of 5-OP-RU (52).

MAIT cells also have a role to play in the progression of diseases relating to metabolic dysfunction. For example, clear and divergent MAIT cell responses in mouse models of type 1 diabetes and obesity are seen: in the former, MAIT cells are protective but in the latter they promote pathogenesis (53). MAIT cell frequency and functional alterations occur during both diseases, although non-obese diabetic (NOD) mice that lacked MR1, and therefore MAIT cells, demonstrated increased gut leakiness and contained higher frequencies of CD8^+^ T cells specific for the islet-specific glucose-6-phosphatase-catalytic-subunit-related protein (53), suggesting a protective role for MAITs. MAIT cells may also promote inflammation in adipose tissue and induce gut dysbiosis in obese mice, the effects of which can be reduced by administration of Ac-6-FP to block their activation via the TCR (54). The exact mechanisms of these divergent roles of MAIT cells remain to be investigated.

Accumulating evidence also posits a role for MAIT cells in viral infection. First, MR1 expression is decreased upon herpes simplex virus type-1 (HSV-1) infection and may function as an immune escape mechanism (55). MAIT cells are also activated during infections with dengue virus, hepatitis C virus and influenza virus in a TCR-independent manner by cytokines such as IFN-γ, type 1 IFNs and IL-18 (56). In addition, degranulation of MAIT cells occurs when co-cultured with macrophages infected with the severe acute respiratory syndrome coronavirus 2 (SARS-CoV-2 (57)). MAIT cell activation was MR1-mediated as it was blocked by anti-MR1 monoclonal antibodies. Such activation was observed when cells were co-incubated for a long period of 4 days, suggesting a slow synthesis of MR1-presented self-antigens and not the presentation of viral antigens *per se*. These results require more work to determine which antigens are presented by MR1 to this population of self-reactive MAIT cells in viral infections.

The role of MAIT cells in diseases outside of bacterial infections is intriguing and increasing evidence suggests that MAIT cells are not limited to recognition of just bacterial antigens but, at least in a few cases, they may also be stimulated by self-antigens. When MAIT cells were originally described, 2 in 20 hybridomas responded towards sterile eukaryotic cell lines (6, 22). Later, MAIT cells were shown to react to MR1-overexpressing HeLa cells under sterile conditions indicating possible reactivity to a self-antigen (4). Further evidence showed reactivity of a MAIT TCR against C1R cells and the possibility that, although very infrequent, MAIT cells exist in the periphery of germ-free mice, suggesting the requirement for positive selection by a self-antigen (24, 58). Thus, the involvement of MAIT cells in a variety of diseases not related to bacterial infection might be mediated by recognition of self-antigen via the TCR. The contribution of cytokines to recognition of self also remains to be defined.

## MR1T cells and therapy

MR1-restricted T cells therefore possess many exciting reactivities of high interest. Our and others’ findings that MR1T cells recognize different tumour cell lines, and the fact that tumour cells expanded *in vivo* also produce self-antigens that stimulate these cells, are of particular interest and have very important implications in designing novel approaches to cell therapy of tumours. The fact that MR1 is oligomorphic and that many tumours stimulate these cells implies that it is possible to exploit MR1T TCRs as off-the-shelf TCRs, ready for immediate use in cell therapy. Whether the observed MR1T cell tumour cross-reactivity is also observed with primary tumour cells growing *in vivo* remains to be investigated; nevertheless, it may represent an important new avenue for tumour cell therapy.

The nature of the tumour antigens stimulating MR1T cells is also a matter of intense investigation in our laboratory. Small metabolites, whose nature remains to be fully characterized, seem to accumulate in transformed and not in healthy cells. Some of these metabolites are associated with cell survival and this could overcome several mechanisms of immune evasion known to date.

In this context, a dysregulated proliferating tumour cell would accumulate metabolites that are otherwise rare or not present in healthy cells. The shift towards an altered metabolome would drive MR1T cell recognition of transformed cells. Such a source of tumour-associated antigens is different from genomic mutations producing neo-peptide antigens, which can be generated in rare cells and not in all types of cancers, even from the same tissue. In these cases, further mutation or loss of such neo-peptide antigens may contribute to the ability of tumour cells to escape an MHC-restricted immune response, granting tumour cell survival. Endogenous metabolites, on the other hand, cannot be mutated to escape immune recognition, and although the metabolic network can be rewired to prevent accumulation of particularly antigenic metabolites, this might result in reduced proliferation or increased apoptosis of the tumour.

## Concluding remarks

The field of MR1-restricted T cells is rapidly expanding and novel aspects of T cell immunity are being revealed at pace. The most striking novel aspects so far are those revealing a preferential role of MAIT cells in tissue homeostasis and their potential involvement in some autoimmune diseases. The identification of MR1T cells also unveils the potential for a large repertoire of MR1-presented antigens that logically function to display the metabolic integrity of cells. As a result, MR1T cells exert potent anti-tumour immunity, independently of the tumour tissue of origin.

Future studies will address the many unknown aspects in MR1 biology, including the mechanisms of bacterial and self-antigens presentation by MR1, how the MR1T TCR repertoire is shaped in the thymus and periphery, whether similar modes of antigen recognition apply to MAIT and MR1T cells, how MR1T antigens are generated and accumulate and in which diseases MR1T cells are important. Regarding these questions, there is significant ground still to be covered but it will likely be discovered rapidly from here.
